# Adolescent self-control behavior predicts body weight through the life course: a prospective birth cohort study

**DOI:** 10.1038/ijo.2015.213

**Published:** 2015-11-03

**Authors:** S Koike, R Hardy, M Richards

**Affiliations:** 1 Medical Research Council Unit for Lifelong Health and Ageing at UCL, London, UK; 2 Office for Mental Health Support, Division for Counseling and Support, The University of Tokyo, Bunkyo-ku, Tokyo, Japan

## Abstract

**Background:**

Weight gain has become one of the biggest issues for healthy aging in middle- and high-income countries. Self-control of emotional reward cues is an important behavioral factor for regulation of weight gain through voluntary diet control and physical activity.

**Methods:**

We tested the associations between teacher-rated self-control at ages 13 and 15 years, and measured body mass index (BMI) between ages 15 and 60–64 years, controlling for confounding factors such as affective symptoms and cognition, using 3873 study members in the Medical Research Council National Survey of Health and Development, also known as the British 1946 birth cohort.

**Results:**

Multivariable regression analysis after adjustment for all covariates showed that lower self-control was associated with higher BMI in all measure points (*P*<0.05). Multilevel modeling using a cubic model showed that there was an association between self-control and BMI at 15 years in females (male: BMI=−0.00 kg m^−2^ per 1 s.d. on the self-control score (95% confidence interval (CI): −0.12 to 0.11), *P* =0.94; female: BMI=−0.27 (−0.42 to −0.11), *P*<0.001). The association became stronger with age in both sexes (BMI=−0.065 (−0.082 to −0.048), *P*<0.001; BMI=−0.036 (−0.057 to −0.015), *P*<0.001). By age 60–64 years, the association between self-control and BMI in men had increased to −0.70 (−0.96 to −0.44) and −0.67 (−1.04 to −0.30) in women.

**Conclusions:**

Lower adolescent self-control was associated with higher BMI through the life course, and this becomes stronger with age. Investigations to test whether intervention to self-control improves obesity are recommended.

## Introduction

Weight gain has become one of the biggest issues for healthy aging in middle- and high-income countries, as rapid lifestyle changes in these countries have led to an increased tendency toward energy-dense diet and decreased physical activity.^1^ Self-control of emotional reward cues^2^ is likely to be important for regulation of weight gain through voluntary diet control and physical activity, as these often require resolving conflict between immediate reward from tempting energy-dense food and from physical relaxation, and reducing these for the longer-term rewarding consequences of weight control.^3^ A well-known developmental model of this is the marshmallow test for children, in which the instant gratification of a small reward (one marshmallow) is pitted against the prospect of a larger reward (more marshmallows) for delayed gratification through waiting.^4^

This self-control emerges in early childhood and matures in adolescence, which is consistent with the developmental timing of hierarchical top–down regulation in the prefrontal cortex.^5, 6^ It then develops into a higher order skill over adulthood, capable of achieving more abstracted goals over a longer period. Previous prospective studies have found that self-control in childhood is associated with a range of outcomes in later life including social, for example, academic and occupational achievement, financial management, family stability and citizenship;^2, 7, 8, 9^ and health related, for example, body mass index (BMI),^10, 11^ metabolic abnormality,^2^ midlife verbal memory,^8^ addictive behavior^2^ and psychotic symptoms.^12^

The present study focused on the outcome of BMI over the life course as a marker of weight gain. Studies testing associations between adolescent self-control and adiposity may be vulnerable to confounding factors such as affective symptoms and cognition, which are both associated with adult BMI,^13, 14, 15, 16^ and which were not consistently controlled in the above previous studies. Furthermore, one previous study used self-reported height and weight, which is subject to systematic bias;^10^ and to the best of our knowledge little is known about adolescent self-control behavior in regard to body weight change over the life course.

The Medical Research Council National Survey of Health and Development (NSHD), also known as the British 1946 birth cohort, offers an excellent opportunity to investigate the relationship between childhood self-control and life course BMI: teachers provided behavioral ratings of study members at ages 13 and 15 years, from which dimensions of self-control, emotionality and conduct problems were derived, and repeated measures of BMI at most sweeps were calculated from measured heights and weights. We hypothesized that lower adolescent self-control is associated with higher and faster increase in BMI over the life course, independently of a range of potential confounders including childhood cognition.

## Materials and methods

### Participants

The NSHD is one of the longest-running prospective large cohort studies, consisting of a social class-weighted sample of 5362 children drawn from all single births within marriage during 1 week in March 1946 in England, Scotland and Wales. The most recent data collection was conducted between 2006 and 2011, when study members were aged 60–64 years. Study members still alive and with a known current address in mainland Britain (*n*=2856) were invited for assessment at one of six clinical research facilities; those unable or unwilling to travel were offered a home visit by a research nurse. A total 2229 participants out of the 2856 invited (78.0% age range=60.3–65.0 years, mean=63.4, s.d.=1.1) underwent assessment: 1690 attended the Clinical Research Facility and the remaining 539 were seen in their homes.^17^ Invitations were not sent to those who had died (*n*=778), who were living abroad (*n*=570), had previously withdrawn from the study (*n*=594) or had been lost to follow-up (*n*=564). All study members participating had their height and weight measured.

A total of 3873 study members had teacher ratings of behavior at both ages 13 and 15 years as well as at least one measure of BMI between ages 15 and 60–64 years (mean number=5.0, s.d.=1.9). Those excluded from analyses had lower birth weight (*P*<0.001), higher weight at age 2 years (*P*=0.009), lower childhood social class (*P*<0.001), lower adult social class (*P*=0.014), and higher BMI at age 15 and lower BMI at age 60–64 years (*P*=0.032 and 0.008, respectively).

Ethical approval for this study was obtained from the Greater Manchester and Scottish committees. All study members gave written informed consent.

### Measures

#### Body mass index

Heights and weights were measured by school doctors at age 15 years and by trained research nurses using standardized protocols at ages 36, 43, 53 and 60–64 years, and by self-reports at ages 20 and 26 years. BMI was calculated at each age using the standard weight/height^2^ formula.

#### Adolescent behavioral problems

Behavior problems in adolescents were rated at age 13 and 15 years by teachers using a forerunner of the Rutter A scale.^18, 19^ These ratings have been classified into three behavioral dimensions reflecting conduct (externalizing) problem (for example, a quarrelsome and aggressive child), emotional (internalizing) problem (for example, extremely fearful) and self-control (for example, a poor worker or lazy).^8^ Each dimension score was calculated from the standardized sum of the factor scores at both ages.^8^

#### Confounding variables

The following childhood variables were treated as potential confounders: birth weight, weight at age 4 years, childhood cognitive ability, family social class at age 11 years (or, if this was unknown, at age 4 or 15 years) and emotional and conduct problems at age 13 and 15 years. Birth weight to the nearest quarter of a pound was extracted from medical records within a few weeks of delivery and converted into kilograms. Weight at 4 years was measured. Childhood cognition was represented as the sum of four tests of verbal and nonverbal ability taken at age 8 (reading comprehension, word reading, vocabulary and picture intelligence) devised by the National Foundation for Education Research.^20^ Father's occupational social class was classified according to the Registrar General and re-grouped into three categories (I or II, III and IV or V).

The following were treated as potential confounders in adulthood: educational attainment at age 26 years and register general head of household occupation social class at age 53 years (three categories, if this was unknown, from age 26 to 43 years). Educational attainment was classified into five categories: none; vocational only; ordinary (O) level, that is, examinations taken at minimum school leaving age; advanced (A) level, that is examinations taken in the final year of secondary education; and higher (degree or equivalent, or beyond).

### Statistical analysis

Multivariable regression analysis was used to test associations between adolescent self-control and BMI at each age, adjusting for potential confounders. Multilevel modeling was then used to model the longitudinal BMI trajectory. This method allows for incomplete outcome data on the assumption of missing at random.^21^ BMI from age 15 to 60–64 years was modeled with linear (age), quadratic (age^2^) and cubic terms (age^3^). Self-control score was added to the model along with emotional problem score as it had a significant association with BMI in our previous study.^15^ Interactions between each personality variable and age were also examined to assess the association with rate of change of BMI.

Statistical significance was set at *P*<0.05. The regression analyses were conducted using SPSS Statistics 22 (IBM, Chicago, IL, USA), and multilevel modeling was performed using ‘nlme' package version 3.1-117 in R version 3.1.1 (The R foundation, Vienna, Austria).^21, 22^

## Results

Mean BMI in both male and female participants increased by age (Table 1). Males had lower BMI at age 15 years and then higher BMI at ages between 20 and 43 years (*P*<0.001), but the difference became non-significant at ages 53 and 60–64 years (Table 1).

### Association of adolescent characteristics with adult BMI

**Figure 1:**
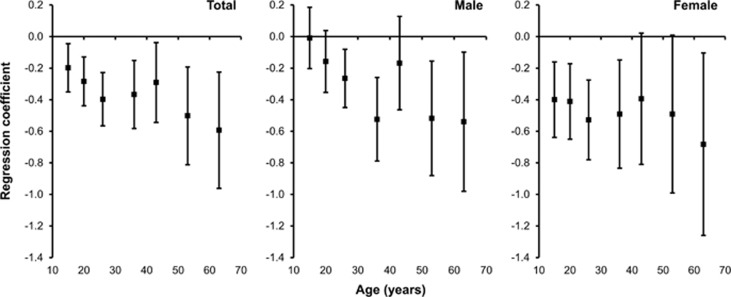
Trajectories in regression coefficient of self-control with BMI. Regression coefficients of self-control considered for potential confounding variables were plotted in total, male and female participants. Bars showed 95% confidence intervals.

The results of multivariable regression analysis to test associations between adolescent self-control and BMI at each age, and results stratified by sex are presented in Table 2. After adjustment for all covariates, which resulted in slight attenuation of the association, lower self-control was associated with higher BMI (*P*<0.05, Figure 1 and Table 2). There was also significant sex × self-control interaction at ages 15, 26 and 36 years (*P*<0.05). When stratified by sex, lower self-control was associated with higher BMI at ages 26, 36, 53 and 60–64 years in males; and at ages 15–36 and 60–64 years in females (*P*<0.05, Figure 1 and Table 2).

### Association with longitudinal BMI trajectory in later life

**Figure 2:**
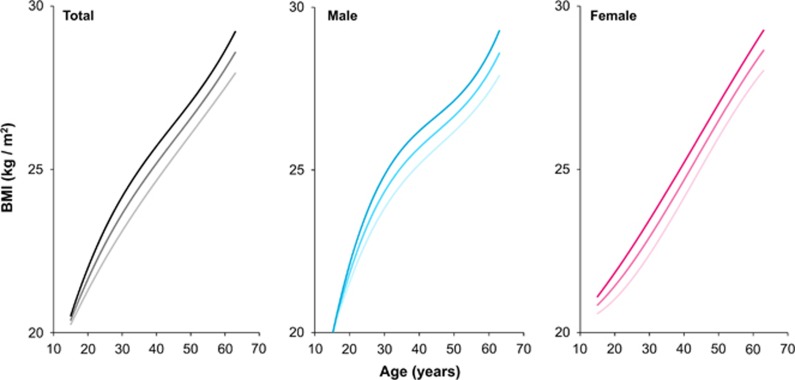
BMI trajectories by self-control. BMI trajectories from age 15 to 60–64 years were shown in total, male and female participants. The trajectories of those with high (mean+s.d.), mean and low (mean−s.d.) self-control for each group were illustrated with pale to dark color gradation.

Multilevel modeling showed that BMI increased significantly with age (*P*<0.001, Table 3 and Figure 2). There was a significant sex × age interaction (*P*<0.001), indicating that BMI in males was lower at age 15 years, and increased faster with age, than in females.

The main effect of self-control was not significant (*P*=0.28, Table 3), but there was a significant sex × self-control interaction (*P*=0.035), indicating a stronger association between self-control and BMI at 15 years in females compared with males (male: BMI=−0.00 kg m^−2^ per 1 s.d. on the self-control score (−0.12 to 0.11), *P*=0.94; female: BMI=−0.27 (−0.42 to −0.11), *P*<0.001; Table 3). The association became stronger with age in both sexes (*P*<0.001), and there was no gender difference in this association (*P*=0.27). By age 60–64 years, the association between self-control and BMI in men had increased to −0.70 (−0.96 to −0.44) kg m^−2^ per unit self-control score and −0.67 (−1.04 to −0.30) in women.

## Discussion

A population representative prospective cohort study revealed that lower self-control was associated with higher BMI, independently of adolescent conduct and emotional problems, early growth and social position, with this association becoming stronger with age. In addition, the association between self-control and BMI emerged earlier in females.

The strengths of this study include the use of a national population-based sample with objective measures of body height and weight over the life course, as well as independent- and prospective-rated behavioral characteristics in adolescence, which are thus not subject to recall bias. The regression analyses were also controlled for several important potential confounding factors in childhood and adulthood. Several limitations should also be considered in this study. Although demographic characteristics in NSHD are still broadly representative of the general source population in the age 60–64 survey,^17^ there was a disproportionate loss to follow-up of those less socially advantaged, although we have no reason to believe that this would have affected the pattern of results. Second, although self-control was assessed from teachers, which is thought to be more objective than ratings from the parents, these ratings could have influenced study member's school performance, to the extent that they reflect teacher–pupil interaction. A mixture of subjective and objective measurements of self-control ability such as delay discounting tasks may help to clarify the relationship between self-control and outcomes.

These results imply that self-control is related to regulatory behavior toward controlling weight, such as dietary control and healthy activity, and this may develop over the life course. There are several possible explanations for the observation that the association strengthens with age. First is the increasing dominance of personal choice with age. Second is the increased availability of energy-dense foods along with increased environmental limitations on routine physical activity experienced by this cohort in adulthood. Thus we would expect self-control to have yet more of an influence on BMI in children who were born after 1946.^23^ Of relevance to this is the fact that food rationing initiated during World War II persisted well into the childhood of NSHD study members. Third, as both higher BMI and lower self-control are associated with lower midlife cognitive function and cognitive decline,^8, 10, 24, 25^ the stronger association with age in this study may be partially mediated by the trajectory of cognitive function in adulthood.

The present study also showed an earlier association between self-control and BMI in females compared with males. There are several potential biological and psychosocial explanations for this. First, as males had lower self-control in adolescence than females, self-control in females may have particular significance to regulation of voluntary diet control and physical activity. Second, associations between self-control and weight change may be stronger among women during perinatal period, which influences postpartum weight retention and in turn may result in increased regulation of dietary and physical activity patterns in those with higher self-control.^26^ Third, although the results were significant after controlling for adult occupational social class, male participants tend to expend more physical activity at work,^27^ which may to some extent limit weight gain regardless of self-control. Fourth, there are also sex differences in neural correlates of reward systems and inhibitory control.^28, 29, 30^ Maturation of self-control occurs in accordance with cognitive regulation of impulsive and reward responses, which is greater in males through adolescence.^29^ The emotional response greatly decreases at the end of adolescence, following which adult males may attain more self-control ability in the face of emotional cues, which might also affect weight control.

In conclusion, a population representative prospective cohort study showed that lower self-control was associated with higher BMI through the life course, with this association becoming stronger with age. Attempts using physical activity and computer training programs in childhood for helping the development of self-control in adolescence may be effective for the performance of inhibition and executive function, as well as real world outcomes such as school performance.^31^ More investigations of effective intervention to efficient maturation of self-control will be needed for better physical and psychosocial outcomes.

## Figures and Tables

**Table 1:** Demographic characteristics in this study

	*Male*		*Female*		P-*value*
	n	*Mean (s.d.)*	n	*Mean (s.d.)*	
*BMI, kg m^−2^*					
15 years	1881	19.6 (2.4)	1700	20.6 (3.0)	<0.001
20 years	1829	22.6 (2.5)	1735	21.8 (2.9)	<0.001
26 years	1822	23.4 (2.8)	1782	22.4 (3.2)	<0.001
36 years	1632	24.8 (3.2)	1648	23.6 (4.1)	<0.001
43 years	1617	25.7 (3.5)	1608	25.2 (4.8)	<0.001
53 years	1452	27.4 (4.0)	1496	27.4 (5.5)	0.81
60–64 years	1061	27.9 (4.1)	1158	27.9 (5.5)	0.92

*Adolescents behavioral problems*^a^					
Self-control	2044	−0.13 (1.01)	1883	0.14 (0.97)	<0.001
Conduct problem	2044	0.11 (0.98)	1883	−0.12 (1.01)	<0.001
Emotional problem	2044	−0.09 (0.96)	1883	0.10 (1.03)	<0.001
Birth weight, g	2798	3444 (558)	2529	3302 (513)	<0.001
Weight at age 2 years, kg	2530	13.2 (1.4)	2303	12.6 (1.4)	<0.001
Cognition at age 8 years^a^	2282	−0.03 (0.88)	2102	0.02 (0.85)	0.070
Childhood social class					0.80
I or II	638		559		
III	1193		1090		
IV or V	621		572		
Educational attainment					<0.001
None	930		835		
Vocational only	141		212		
O level	343		520		
A level	590		450		
Higher	304		107		
Occupational social class at age 53 years					<0.001
I or II	1015		674		
III	857		937		
IV or V	272		445		

Abbreviation: BMI, body mass index.

a *Z*-scores.

**Table 2:** The association of self-control with BMI at each age

*Age*	*Total crude*	*Adjusted model*	*Male crude*	*Adjusted model*	*Female crude*	*Adjusted model*
15 Years	0.03 (−0.06 to 0.13)	−0.20 (−0.35 to −0.04)*	0.10 (−0.02 to 0.21)	−0.01 (−0.20 to 0.18)	−0.19 (−0.35 to −0.04)*	**−0.40 (−0.64 to −0.16)**^**†**^
20 Years	**−0.30 (−0.39 to −0.20)**^**‡**^	**−0.28 (−0.44 to −0.13)**^**‡**^	**−0.16 (−0.28 to −0.04)**^**†**^	−0.16 (−0.35 to 0.04)	**−0.35 (−0.50 to −0.20)**^**‡**^	**−0.41 (−0.65 to −0.17)**^**‡**^
26 Years	**−0.51 (−0.62 to −0.40)**^**‡**^	**−0.40 (−0.57 to −0.22)**^**‡**^	**−0.35 (−0.49 to −0.21)**^**‡**^	−0.27 (−0.49 to −0.04)*	**−0.55 (−0.72 to −0.38)**^**‡**^	**−0.53 (−0.78 to −0.27)**^**‡**^
36 Years	**−0.57 (−0.71 to −0.42)**^**‡**^	**−0.37 (−0.58 to −0.15)**^**‡**^	**−0.40 (−0.57 to −0.23)**^**‡**^	−0.28 (−0.55 to −0.01)*	**−0.58 (−0.80 to −0.36)**^**‡**^	−0.44 (−0.78 to −0.10)*
43 Years	**−0.57 (−0.73 to −0.41)**^**‡**^	−0.29 (−0.55 to −0.04)*	**−0.42 (−0.60 to −0.23)**^**‡**^	−0.17 (−0.46 to 0.13)	**−0.67 (−0.94 to −0.40)**^**‡**^	−0.39 (−0.81 to 0.02)
53 Years	**−0.63 (−0.82 to −0.43)**^**‡**^	−0.50 (−0.81 to −0.19)*	**−0.58 (−0.80 to −0.35)**^**‡**^	**−0.52 (−0.88 to −0.15)**^**†**^	**−0.71 (−1.03 to −0.38)**^**‡**^	−0.49 (−0.99 to 0.01)
60–64 Years	**−0.72 (−0.95 to −0.49)**^**‡**^	**−0.59 (−0.96 to −0.23)**^†^	**−0.75 (−1.02 to −0.48)**^**‡**^	−0.54 (−0.98 to −0.10)*	**−0.74 (−1.12 to −0.37)**^**‡**^	−0.68 (−1.26 to −0.10)*

BMI (95% confidence interval). Bold showed significant coefficient (*P<0.05, ^†^*P*<0.01, ^‡^*P*<0.001).

Adjusted model is adjusted for sex, birth weight, weight at age 4 years, overall cognition at age 8 years, childhood social class, conduct and emotional problem at ages 13 and 15 years, educational attainment, and head of household occupational social class at age 53 years.

**Table 3:** Multilevel modeling for adolescent self-control and BMI through the life course

*Independent variables*	*Total (*n=*3873), BMI (95% CI)*	P-*value*	*Male (*n=*2015), BMI (95% CI)*	P-*value*	*Female (*n=*1858), BMI (95% CI)*	P-*value*
(Intercept)	**18.9** **(18.6 to 19.2)**	**<0.001**	**19.9** **(19.8 to 20.0)**	**<0.001**	**20.9** **(20.7 to 21.0)**	**<0.001**
Sex	**0.98** **(0.80 to 1.16)**	**<0.001**				
Age	**0.75** **(0.71 to 0.79)**	**<0.001**	**0.43** **(0.41 to 0.45)**	**<0.001**	**0.11** **(0.09 to 0.13)**	**<0.001**
Age^2^	**−0.025** **(−0.027 to −0.023)**	**<0.001**	**−0.011** **(−0.012 to −0.010)**	**<0.001**	**0.003** **(0.002 to 0.004)**	**<0.001**
Age^3^ (× 10^−4^)	**2.8** **(2.5 to 3.0)**	**<0.001**	**1.2** **(1.1 to 1.4)**	**<0.001**	**−0.3** **(−0.4 to −0.2)**	**<0.001**
Sex × age	**−0.32** **(−0.35 to −0.29)**	**<0.001**				
Self-control	0.13 (−0.14 to 0.40)	0.28	−0.00 (−0.12 to 0.11)	0.94	**−0.27** **(−0.42 to −0.11)**	**<0.001**
Sex × self-control	**−0.19** **(−0.36 to −0.01]**	**0.035**				
Age × self-control	**−0.059** **(−0.077 to −0.040)**	**<0.001**	**−0.065** **(−0.082 to −0.048)**	**<0.001**	**−0.036** **(−0.057 to −0.015)**	**<0.001**
Sex × age × self-control	0.005 (−0.004 to 0.013)	0.27				

Abbreviation: BMI, body mass index.

Significant coefficients were shown in bold (*P*<0.05). BMI at age 15 years was set as reference. Male was coded as 1 and female as 2. These models also include other independent variables such as emotional problem, and sex × age^2^, sex × age^3^, sex × emotional problem, age × emotional problem, age^2^ × self-control, age^3^ × self-control, and sex × age × emotional problem interactions.
